# Photobiomodulation for skeletal muscle regeneration: mechanisms, dosimetry, and translational challenges

**DOI:** 10.3389/fbioe.2026.1921612

**Published:** 2026-09-18

**Authors:** Ryan Ahmed, Kevin L. Zhang, Rabab Hamzah, N. Sertac Artan, Karrer Alghazali

**Affiliations:** 1 Department of Electrical and Computer Engineering, New York Institute of Technology, New York, NY, United States; 2 Tissue Engineering and Advanced Materials (TEAM) Laboratory, Department of Electrical and Computer Engineering, College of Engineering and Computing Sciences, New York Institute of Technology, Old Westbury, NY, United States; 3 College of Osteopathic Medicine, New York Institute of Technology, Old Westbury, NY, United States; 4 Department of Biological and Chemical Sciences, New York Institute of Technology, New York, NY, Unites states

**Keywords:** artificial intelligence, cytochrome c oxidase, dosimetry, light transport, low-level laser therapy, mitochondria, Monte Carlo simulation, muscle regeneration

## Abstract

Photobiomodulation (PBM) has gained increasing attention as a non-invasive modality for enhancing skeletal muscle recovery; however, its clinical translation remains limited by inconsistencies in dosimetry, methodology, and reporting across studies. This review synthesizes over two decades of preclinical and clinical research to evaluate how key parameters such as wavelength, irradiance, fluence, pulse structure, and timing affect muscle regeneration. Evidence indicates that red and near-infrared light modulate mitochondrial cytochrome c oxidase activity, promoting nitric oxide dissociation, increasing ATP production, and inducing controlled reactive oxygen species (ROS) signaling that supports satellite cell activation, angiogenesis, and anti-inflammatory responses. These effects follow a biphasic dose–response relationship and are strongly influenced by tissue optical properties, which differ significantly between animal models and humans, contributing to variability in clinical outcomes. This review highlights critical challenges, including uncertainties in light delivery and tissue-level dose deposition. This review outlines recommendations to improve reproducibility, including standardized dosimetry, radiometric calibration, and integration of PBM with exercise-based rehabilitation. Emerging approaches, such as Monte Carlo light transport modeling and patient-specific optical simulations, are discussed as promising strategies for personalized treatment planning. Collectively, these advances support the transition of PBM from an empirical intervention toward a mechanistically guided and clinically reliable therapy for skeletal muscle regeneration.

## 1 Contextualizing photobiomodulation in muscle regeneration

Photobiomodulation (PBM), also known as low-level laser therapy (LLLT), is a promising non-invasive modality for enhancing skeletal muscle repair, reducing post-exercise inflammation, and accelerating recovery. PBM utilizes monochromatic or narrow-band light (typically 630–980 nm) to stimulate mitochondrial pathways and downstream cellular repair processes without inducing thermal damage (Hamblin, 2017; Leal-Junior, 2015). Over the past two decades, PBM has been increasingly used in clinical and sports medicine, including for delayed-onset muscle soreness and post-surgical or traumatic rehabilitation (Oliveira et al., 2023; Assis et al., 2015). However, its integration into standardized clinical protocols remains limited due to variability in outcomes across studies using similar models (Tunér and Jenkins, 2016; Carroll et al., 2008). Although numerous experimental and clinical studies have reported beneficial effects of PBM on skeletal muscle repair, considerable variability exists in treatment outcomes owing to differences in irradiation parameters, tissue characteristics, and study design. Consequently, translating promising laboratory findings into standardized clinical practice remains a significant challenge.

A primary source of inconsistency in PBM research is the lack of standardization in treatment parameters. PBM efficacy depends on parameters such as wavelength, irradiance, energy density, pulse frequency, and timing relative to injury or exercise (Carroll et al., 2008; Huang et al., 2011). Studies using near-infrared (810–980 nm) light penetrate deeper into tissue and result in greater mitochondrial activation than shorter red wavelengths (630–660 nm) (Wong-Riley et al., 2005; Sabino et al., 2016). However, standardized protocols for optimal parameter combinations remain undeveloped. The biphasic dose–response phenomenon, first described by Arndt and Schulz and later confirmed by Huang et al. (2011) and Nie et al. (2023), indicates that low doses may be sub-therapeutic, whereas doses above ∼10 J/cm^2^ may inhibit regeneration. Carroll’s three-dimensional dose model (Carroll et al., 2008) demonstrated that beam geometry and tissue optical coefficients determine whether responses are biostimulatory or inhibitory, highlighting the importance of spatially resolved dosimetry.

This issue is further compounded by the methodological heterogeneity across PBM studies. PBM trials vary in target muscles, delivery geometry, tissue depth, and outcome measures. Additionally, critical parameters such as spot size and power density are frequently omitted (Tunér and Jenkins, 2016). Systematic reviews have shown that many studies fail to report sufficient dosimetry information to enable replication of treatment protocols (Tunér and Jenkins, 2016; Shivappa et al., 2025). As a result, meta-analyses of exercise-induced muscle damage report both significant and null effects under similar conditions (Oliveira et al., 2023). This lack of replication has perpetuated debate over whether differences in PBM outcomes arise from biological variability or inconsistent experimental design. Collectively, these findings suggest that inconsistencies in PBM outcomes cannot be attributed solely to biological variability. Rather, they arise from the interaction between heterogeneous treatment parameters, differences in tissue optical properties, and inconsistent experimental methodology. Consequently, improving reproducibility will require greater standardization of dosimetry, reporting practices, and study design across both preclinical and clinical investigations.

Mechanistic studies indicate that PBM primarily acts via activation of cytochrome c oxidase (CCO), increasing ATP production and modulating reactive oxygen species (ROS) and nitric oxide (NO) signaling (Hamblin, 2017; Hamblin, 2018; Quirk and Whelan, 2020; Poyton and Ball, 2011). These downstream effects promote satellite cell proliferation, angiogenesis, and anti-inflammatory responses, contributing to muscle regeneration (Anderson, 2000; Khorsandi et al., 2020). These processes depend on photon absorption within CCO chromophore bands, emphasizing the need for wavelength optimization specific to the application (Gong et al., 2021; Lalonde et al., 2020). Taken together, these mechanistic findings indicate that PBM exerts multifactorial biological effects rather than acting through a single molecular pathway. While cytochrome c oxidase remains the most widely accepted primary photoreceptor, increasing evidence suggests that tissue-specific optical properties, wavelength-dependent interactions, and additional signaling pathways may influence therapeutic outcomes. This complexity likely contributes to the variability reported across experimental and clinical studies.

Importantly, no single dosimetric parameter determines therapeutic success. Instead, biological responses arise from the combined influence of wavelength, irradiance, fluence, treatment duration, pulse characteristics, tissue composition, and injury stage. Failure to consistently report and standardize these interacting variables has become one of the principal barriers to comparing studies and establishing standardized clinical protocols. Given these issues, a major limitation is the lack of standardized dosimetry and timing frameworks in PBM therapy for skeletal muscle recovery. Collectively, current evidence suggests that PBM enhances mitochondrial bioenergetics, reduces oxidative stress, and promotes tissue regeneration within specific therapeutic parameter ranges. Accordingly, this narrative review critically examines current evidence on PBM for skeletal muscle regeneration by integrating biological mechanisms, dosimetric principles, computational modeling, and clinical studies. Particular emphasis is placed on methodological variability, reproducibility challenges, and emerging computational approaches that may facilitate personalized treatment planning. By synthesizing evidence across these disciplines, this review aims not only to summarize existing knowledge but also to identify critical barriers, unresolved controversies, and future research priorities that must be addressed before PBM can achieve broader clinical translation (Shivappa et al., 2025).

## 2 Literature search strategy

This article was conducted as a narrative review of the literature to provide a comprehensive overview of current developments in photobiomodulation (PBM) for skeletal muscle regeneration. Literature searches were performed using PubMed, Scopus, Web of Science, and Google Scholar to identify peer-reviewed studies published primarily between January 2000 and March 2025, while seminal publications predating this period were included where historically relevant. Search terms included combinations of “photobiomodulation”, “low-level laser therapy”, “skeletal muscle”, “muscle regeneration”, “mitochondria”, “cytochrome c oxidase”, “dosimetry”, “Monte Carlo simulation”, “light transport”, and “artificial intelligence”. Additional references were identified through manual review of bibliographies from key review articles.

Studies were selected based on their relevance to the review objectives, including skeletal muscle regeneration, PBM mechanisms, dosimetry, computational modeling, and clinical translation. Both experimental and clinical investigations were considered, together with systematic reviews that provided mechanistic or methodological insights. Conference abstracts, non-peer-reviewed publications, duplicate reports, and studies unrelated to musculoskeletal photobiomodulation were excluded. Because the objective of this article was to critically synthesize multidisciplinary evidence rather than quantitatively pool outcomes, a narrative review methodology was considered the most appropriate approach.

## 3 Methodological evolution in photobiomodulation research

Systematic reviews and meta-analyses provide insight into the evolution of photobiomodulation (PBM) research over the past two decades. The progression from single-wavelength laser systems to LED-based, pulsed, and multimodal PBM reflects technological advancement rather than methodological maturity. Although newer devices provide greater flexibility in treatment delivery, they have also introduced additional variables that complicate cross-study comparisons. Consequently, improvements in PBM technology have not been matched by equivalent progress in protocol standardization, making it difficult to determine whether differences in reported outcomes arise from device characteristics or true biological effects. A major review by Oliveira et al. (2023) highlighted significant inconsistencies in inclusion criteria, search strategies, and outcome measures across PBM studies targeting skeletal muscle recovery. Early studies focused on single-wavelength, continuous-wave laser systems, whereas more recent work includes LED-based, pulsed, and multimodal systems, increasing variability in study design without improving standardization.

Most systematic reviews, including those by Kulkarni et al. (2022) and González-Muñoz et al. (2023), use major databases such as PubMed, Scopus, and Web of Science with keywords including “photobiomodulation,” “low-level laser therapy,” and “muscle recovery,” but apply highly variable inclusion criteria. For example, Oliveira et al. (2023) and Ferraresi et al. (2016) restricted inclusion to studies on skeletal muscle in both animal and human models. Other umbrella reviews, such as Son et al. (2025), extend inclusion beyond musculoskeletal conditions to neurological and inflammatory disorders. This methodological variability complicates cross-study comparisons and limits the ability to identify optimal therapeutic parameters. Importantly, variability in review methodology affects not only the interpretation of individual studies but also the conclusions drawn regarding PBM efficacy. Reviews that combine heterogeneous patient populations, injury models, treatment protocols, and outcome measures may obscure clinically meaningful dose-response relationships. Therefore, future evidence syntheses should emphasize clinically comparable populations and standardized reporting frameworks rather than simply increasing the number of included studies.

Bias in both experimental and clinical PBM studies represents an additional challenge. Farahani et al. (2007) investigated design-related bias in clinical research and emphasized that small sample sizes and inadequate blinding can distort reported outcomes. Similar methodological limitations are evident in PBM research. Grimes (2025) also emphasized that visible light therapy studies often lack standardized, radiometrically validated protocols, leading to discrepancies between reported irradiance and the actual delivered dose. Tunér and Jenkins (2016) found that fewer than one-third of PBM trials report complete information on power density, beam area, or fluence, limiting reproducibility.

Methodological issues are also observed in non-muscle applications. Chen K.-Y. et al. (2025) and Rassi et al. (2024) reported inconsistent dosimetry and lack of standardized safety protocols in ophthalmological PBM studies. Similarly, Fantaguzzi et al. (2023) found that many multi-wavelength studies lack clear justification for wavelength selection and rely on arbitrary combinations. Collectively, these findings indicate that translational challenges in PBM are not limited to skeletal muscle but reflect broader issues in biomedical photonics research.

Emerging methodological approaches aim to address these inconsistencies. Ferraresi et al. (2016) advocate the use of objective functional outcomes, such as muscle strength and oxygen saturation, rather than subjective pain measures. Kumar et al. (2024) extend this approach by combining PBM with prescribed exercise programs, improving reproducibility compared to PBM alone. Reviews across multiple domains, including Son et al. (2025), recommend adopting standardized reporting templates similar to CONSORT guidelines to ensure reproducibility and traceability to international radiometric calibration standards. Collectively, literature published between 2010 and 2025 demonstrates gradual but incomplete progress toward improving methodological rigor in PBM research. Although systematic reviews, improved outcome measures, and advances in LED technologies and computational modeling have strengthened the evidence base, persistent deficiencies in dosimetric reporting, protocol standardization, multicenter validation, and methodological transparency continue to limit reproducibility. Addressing these challenges will require coordinated efforts among researchers, clinicians, device manufacturers, and journal editors to establish consensus reporting standards and harmonized treatment protocols. Until these foundational issues are resolved, variability in study design will continue to hinder reliable cross-study comparisons and widespread clinical translation of PBM.

## 4 Mechanisms of photobiomodulation in skeletal muscle

Photobiomodulation (PBM) in skeletal muscle involves a series of photochemical and biochemical processes that regulate mitochondrial metabolism, nitric oxide (NO) signaling, and stem cell activation. A widely accepted molecular target of PBM is cytochrome c oxidase (CCO; complex IV of the mitochondrial electron transport chain), which contains copper and heme chromophores that absorb photons in the red to near-infrared spectrum (600–950 nm) (Hamblin, 2017; Hamblin, , 2018; Quirk and Whelan, 2020; Poyton and Ball, 2011). Photon absorption at the CuB binuclear center promotes photodissociation of inhibitory nitric oxide (NO), thereby enhancing electron transport and increasing the efficiency of proton pumping across the mitochondrial membrane (Quirk and Whelan, 2020; Poyton and Ball, 2011). This process increases mitochondrial membrane potential (ΔΨm), enhances ATP production, and modulates reactive oxygen species (ROS) generation, collectively influencing gene expression and promoting cellular repair processes (Lalonde et al., 2020; Quirk and Whelan, 2021; Fallahi et al., 2023). PBM-induced changes in cellular redox state not only support energy production but also regulate cellular signaling pathways (Anderson, 2000; Pope et al., 2020). Sublethal increases in mitochondrial ROS activate transcription factors such as NF-κB and AP-1, promoting the expression of growth factors and cytokines involved in muscle regeneration. Although mitochondrial activation through cytochrome c oxidase (CCO) remains the most widely accepted explanation for PBM, accumulating evidence indicates that its biological effects are unlikely to result from a single photoreceptor or signaling pathway. Instead, PBM appears to initiate a network of interconnected biochemical and biophysical responses that vary according to wavelength, irradiance, tissue composition, and physiological state, as depicted in Figure 1. This emerging perspective highlights the complexity of PBM and underscores the importance of considering multiple complementary mechanisms when interpreting therapeutic outcomes.

**FIGURE 1:**
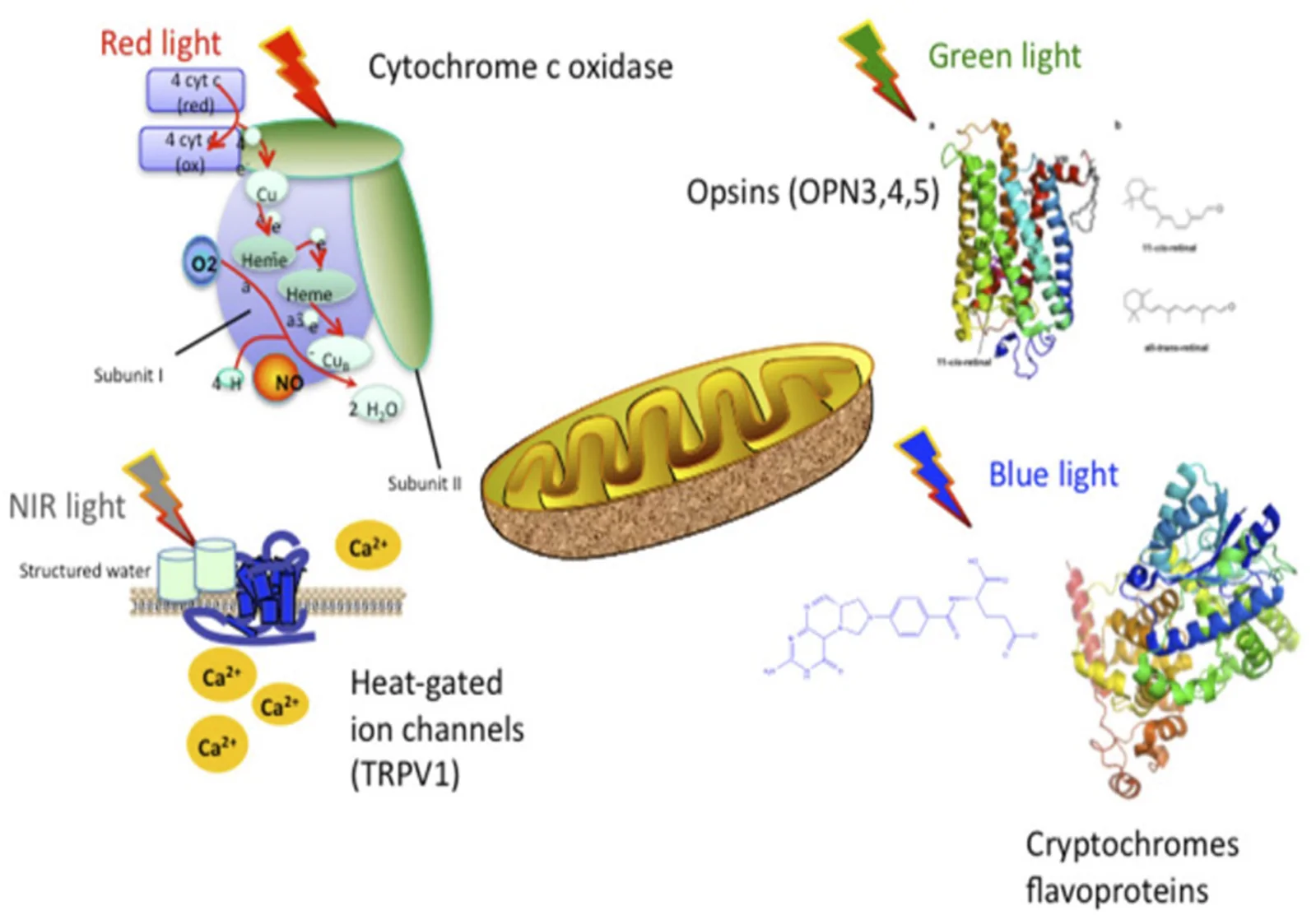
Major chromophores and photoreceptors implicated in photobiomodulation. Cytochrome c oxidase absorbs primarily red and near-infrared light through heme and copper centers; TRP ion channels may respond to NIR and blue light through structured water; opsins absorb blue/green light through cis-retinal; and flavoproteins/cryptochromes absorb mainly blue light. Adapted from Hamblin (2017). This figure provides the mechanistic foundation for understanding how wavelength-dependent interactions with specific chromophores initiate the biological processes underlying photobiomodulation.

PBM-induced nitric oxide release promotes vasodilation, improves microcirculation, and supports satellite cell activation, all of which are critical for myogenesis (Anderson, 2000). Anderson (2000) demonstrated that nitric oxide acts as a key signaling molecule enhancing satellite cell proliferation during muscle regeneration. Beyond mitochondrial effects, PBM also influences stem cell behavior and enhances the regenerative capacity of progenitor cells. In this context, Khorsandi et al. (2020) demonstrated that exposure of mesenchymal and adipose-derived stem cells to near-infrared light increases proliferation, enhances mitochondrial activity, and reduces apoptotic signaling. These findings suggest that PBM enhances endogenous regeneration by creating a metabolic environment that supports cell survival and differentiation. Fallahi et al. (2023) reported similar improvements in mitochondrial activity in diabetic adipose-derived stem cells, suggesting that PBM may mitigate metabolic deficits in pathological conditions. At the cellular network level, PBM also modulates nitric oxide synthase (NOS) activity and downstream signaling pathways involved in angiogenesis and inflammation. Leyane et al. (2021) reported that PBM modulates TGF-β and VEGF signaling in wound healing models, with implications for skeletal muscle repair. Controlled ROS production contributes to redox signaling pathways that regulate extracellular matrix remodeling and fibroblast differentiation without inducing oxidative damage (Lalonde et al., 2020; Pope et al., 2020). Importantly, ROS generated during PBM exhibit a biphasic biological response. Moderate increases in ROS act as essential secondary messengers that regulate cellular adaptation, proliferation, and tissue repair, whereas excessive ROS accumulation may promote oxidative stress and cellular injury. This dose-dependent balance further emphasizes the importance of optimizing PBM dosimetry to maximize therapeutic signaling while minimizing adverse biological effects. Cytochrome-specific analyses provide further insight into the molecular mechanisms underlying PBM. Quirk and Whelan (2021) tested whether red to near-infrared irradiation and nitric oxide donor exposure altered oxygen consumption in isolated cytochrome c oxidase. Their findings did not show a significant light-induced increase in oxygen consumption and suggest that the CCO/NO mechanism remains mechanistically debated. Lalonde et al. (2020) and Pope et al. (2020) extended this hypothesis by finding that low irradiance visible light differentially alters the activity of key enzymes in the mitochondrial electron transport chain, enhancing mitochondrial efficiency without inducing photothermal stress. Collectively, these studies suggest that PBM modulates mitochondrial redox homeostasis and nitric oxide–mediated signaling pathways involved in skeletal muscle regeneration. This evidence supports a dual functional role for PBM: restoring bioenergetic balance through CCO activation while activating paracrine and autocrine signaling pathways that promote angiogenesis, anti-inflammatory responses, and stem cell recruitment. These mechanisms underpin the biological effects observed in both preclinical and clinical studies of muscle recovery. Taken together, these mechanistic studies indicate that PBM exerts pleiotropic biological effects rather than acting through a single pathway. While mitochondrial activation remains the most widely accepted mechanism, increasing evidence suggests that treatment responses are influenced by wavelength-specific photoreceptors, tissue optical properties, and the physiological state of the target tissue. These interconnected mechanisms are summarized in Figure 2 and illustrate how mitochondrial activation, redox signaling, and downstream regenerative pathways converge to promote skeletal muscle repair. Together, they also highlight the biological complexity that contributes to variability across experimental and clinical investigations.

**FIGURE 2:**
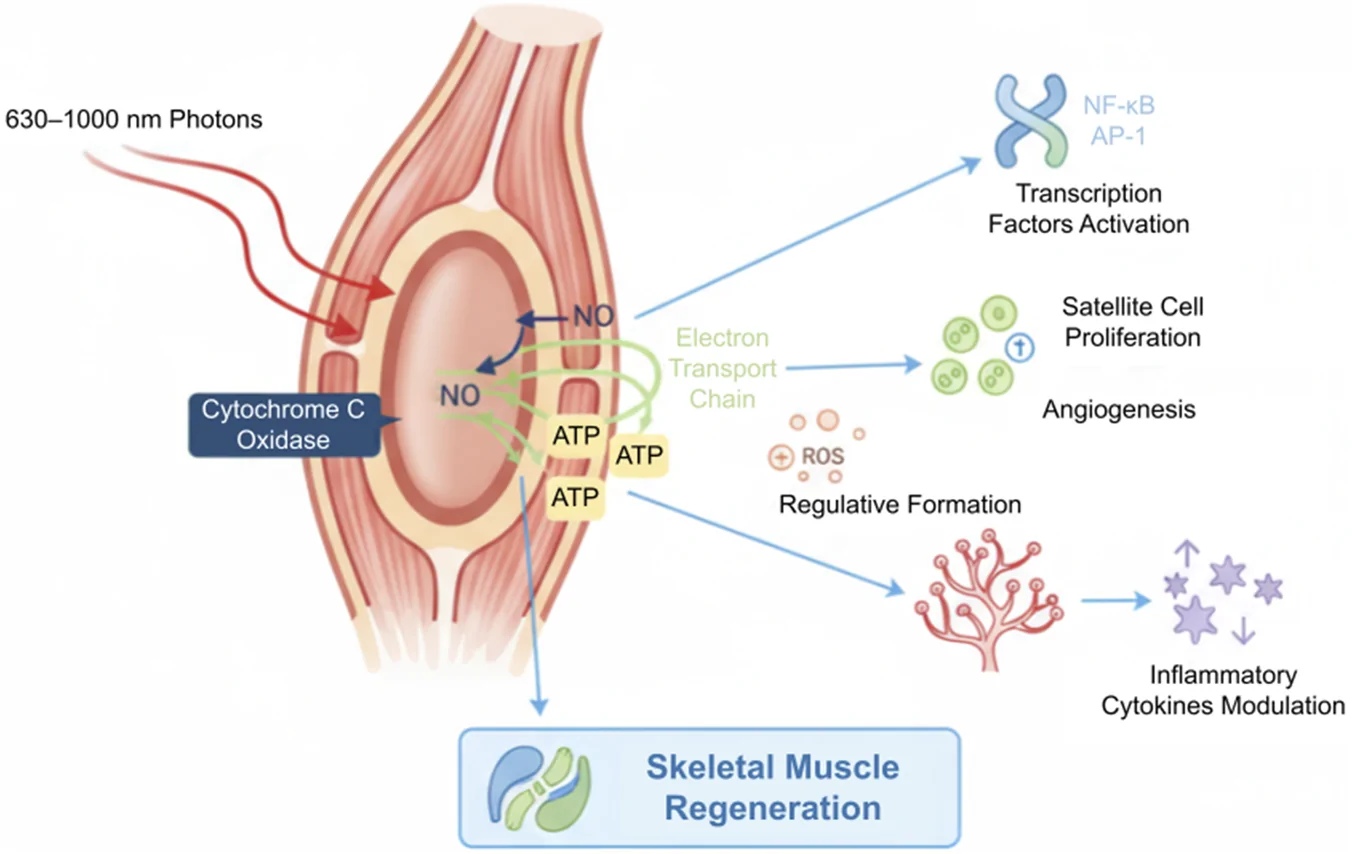
Mechanism of Photobiomodulation in Skeletal Muscle. Red to near-infrared light (630–1000 nm photons) penetrates skeletal muscle tissue and is absorbed by cytochrome c oxidase (CCO) within the mitochondrial respiratory chain. Photon absorption displaces nitric oxide (NO) from CCO, enhancing electron transport efficiency and leading to increased mitochondrial membrane potential, ATP synthesis, and controlled reactive oxygen species (ROS) signaling. These biochemical changes activate downstream pathways that promote (i) transcription factor activation (NF-κB, AP-1), (ii) satellite cell proliferation and angiogenesis, (iii) ROS-mediated regulatory signaling, and (iv) modulation of inflammatory cytokine expression. Collectively, these responses converge to enhance skeletal muscle regeneration and repair following injury or oxidative stress.

### 4.1 Emerging alternative photoreceptors and signaling pathways

Despite the substantial body of evidence supporting CCO as the primary intracellular chromophore for red and near-infrared light, several questions remain unresolved. Experimental findings are not always consistent across wavelengths, tissue types, or disease models, suggesting that CCO activation alone may not fully explain the diverse biological responses reported in the literature. These inconsistencies have prompted increasing interest in complementary photoreceptive mechanisms that may operate independently or synergistically with mitochondrial signaling.

Although cytochrome c oxidase remains the predominant mechanistic model, increasing evidence suggests that PBM responses arise from multiple complementary photochemical and photophysical pathways rather than a single intracellular target. Recent investigations have therefore proposed several additional photoreceptors and biophysical mechanisms that may contribute to therapeutic outcomes.

Transient receptor potential (TRP) channels, particularly members of the TRPV family, have emerged as potential mediators of PBM by regulating calcium influx following light exposure. Calcium signaling plays a central role in cell proliferation, migration, differentiation, and inflammatory regulation, suggesting that TRP channel activation may contribute to many downstream regenerative effects traditionally attributed solely to mitochondrial stimulation.

Flavoproteins and cryptochromes have likewise been proposed as photoresponsive molecules capable of absorbing blue and green wavelengths, thereby influencing intracellular redox signaling and circadian regulatory pathways. Although their contribution to skeletal muscle regeneration remains less well established than that of CCO, these photoreceptors may help explain wavelength-dependent biological responses observed in experimental studies.

Another emerging hypothesis involves the interaction of light with interfacial or structured water surrounding cellular proteins and membranes. According to this model, PBM may alter the physical organization of intracellular water layers, thereby influencing protein conformation, enzyme kinetics, ion transport, and mitochondrial efficiency independently of classical chromophore absorption. While experimental support for this mechanism remains limited, it illustrates the growing recognition that PBM likely involves both photochemical and photophysical processes.

Collectively, these alternative mechanisms do not diminish the importance of cytochrome c oxidase but instead suggest that PBM operates through multiple interconnected pathways whose relative contributions depend upon wavelength, tissue optical properties, cellular metabolic state, and treatment parameters. Future mechanistic investigations integrating molecular biology with advanced optical modeling will be essential for resolving these relationships.

## 5 Key parameters affecting outcomes

Precise control of key PBM parameters, including wavelength, intensity, power density, pulse duration, and timing, is critical for therapeutic effectiveness. Lack of standardization in these parameters contributes to variability across studies and underlies the biphasic dose–response relationship described by Huang et al. (2011), Karu et al. (2005), and Karu et al. (2004). Appropriate parameter selection enables activation of photoreceptors such as cytochrome c oxidase (CCO) without inducing photothermal or excessive oxidative stress, which could suppress mitochondrial function.

Differences in tissue absorption and scattering coefficients between red (630–660 nm) and near-infrared (NIR; 810–980 nm) light determine their suitability for superficial versus deep tissue applications, as depicted in Figure 3. Rather than representing universally superior treatment options, different wavelengths should be viewed as complementary therapeutic tools whose effectiveness depends on tissue depth, optical properties, mitochondrial density, and the clinical objective of treatment. Enwemeka (2000) showed that 632.8 nm light is absorbed primarily by the epidermis and superficial fascia, whereas 904 nm light penetrates several millimeters into soft tissue with minimal attenuation. An optical model developed by Kaub and Schmitz (2023) demonstrated that 905 nm and 1064 nm wavelengths achieve greater tissue penetration than shorter wavelengths due to reduced hemoglobin absorption. Hudson et al. (2013) measured the transmission of 808 nm and 980 nm radiation through bovine muscle and subcutaneous tissue, suggesting that these wavelengths are among the most effective for deep muscle treatment.

**FIGURE 3:**
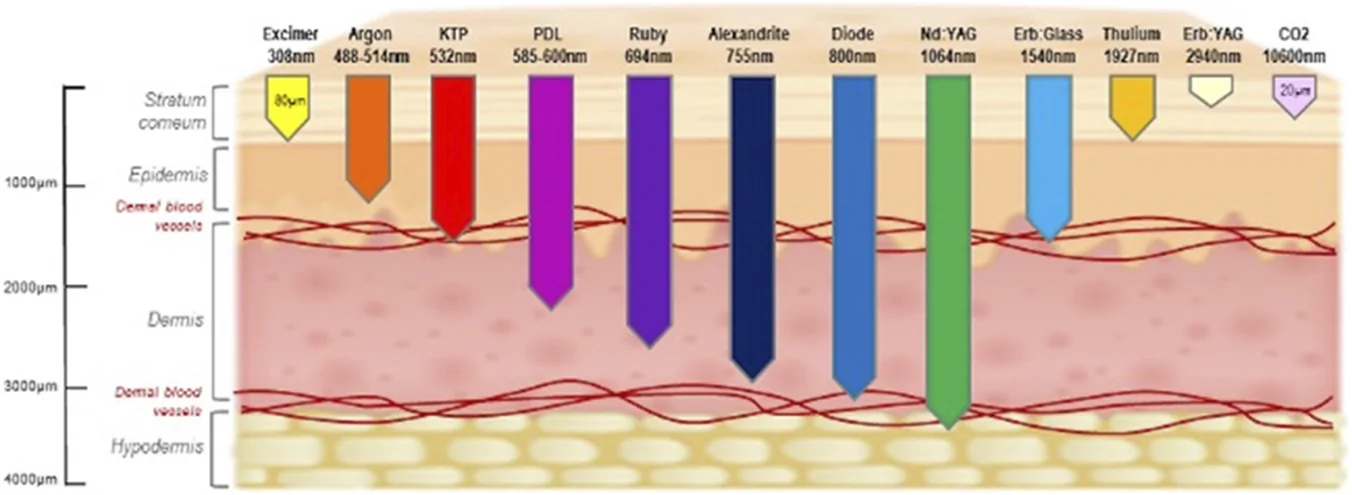
Tissue penetration depths of commonly used therapeutic laser wavelengths. Visible and near-infrared wavelengths differ substantially in absorption and scattering across skin and soft tissue layers, with longer near-infrared wavelengths generally achieving deeper penetration. Adapted from Park et al., (2024); figure courtesy of the Wellman Center for Photomedicine. These penetration characteristics provide the dosimetric rationale for selecting near-infrared wavelengths (approximately 810–980 nm) when targeting deeper skeletal muscle tissues, whereas shorter red wavelengths are generally more appropriate for superficial structures (Park et al., 2024).

While Karu et al. (2005) demonstrated that both red and NIR light reduce CCO in monolayer cell culture, longer wavelengths may activate mitochondria more efficiently due to improved photon absorption. Furthermore, Assis et al. (2016) demonstrated wavelength-specific biological responses: red light was associated with faster resolution of inflammation, whereas NIR promoted greater myofiber regeneration in animal models. These studies provide the basis for selecting wavelengths based on the depth and metabolic demand of the target tissue.

Fluence (energy delivered per unit area, J/cm^2^) is a measure of the total number of photons delivered to the target tissue. However, the biological effect of PBM follows a nonlinear, biphasic relationship. van Breugel and Bär (1992) demonstrated that biostimulatory effects occur at fluences between 3 and 6 J/cm^2^, whereas higher fluences inhibit cellular activity. Karu et al. (2004) proposed that the inhibition was due to transient mitochondrial over-reduction, which leads to feedback downregulation of the electron transport chain. Both power density and exposure time determine fluence; however, they must remain below thresholds that induce thermal accumulation. Importantly, fluence should not be interpreted independently of irradiance or treatment duration, as identical fluence values can be achieved using markedly different parameter combinations that may produce distinct biological responses. This interaction underscores the need to report complete dosimetric protocols rather than isolated energy values.

The temporal characteristics of PBM influence its interaction with cellular metabolic processes. Intermittent stimulation reduces cellular fatigue and sustains mitochondrial responsiveness in both electrical and optical analogues of PBM. Kesar and Binder‐Macleod (2006) demonstrated that pulse width and duration influence recovery between excitation events, analogous to the effects of pulsed PBM on ATP production. Albuquerq et al. (2015) showed that pre-irradiation parameters influence CCO activity.

The timing of PBM relative to the onset of injury or exertion significantly influences treatment outcomes. Liu et al. (2009) showed that post-exertional irradiation reduces markers of oxidative stress and inflammation compared to delayed treatment, indicating a limited therapeutic window. Recent advancements in transcutaneous and implantable PBM systems enable precise energy delivery to targeted tissues. Stevens et al. (2024) demonstrated functional neuroregeneration using implantable light-emitting devices that deliver consistent fluences to heterogeneous tissue, a concept that is potentially translatable to deep muscle repair. Optical coupling via biodegradable scaffolds or polymeric waveguides provides additional control over intramuscular photon distribution and aligns with emerging computational modeling approaches.

In summary, PBM efficacy arises from a complex interaction between optical parameters and tissue biophysical properties. Reproducibility depends on accurate reporting of key parameters, including wavelength, power density, beam area, and timing, which are frequently underreported in the literature. Standardizing these parameters is essential for advancing PBM from an empirical modality to a precision-based therapeutic approach. Future progress toward precision PBM will depend not only on improved device engineering but also on internationally standardized reporting guidelines, computational treatment planning, and patient-specific dosimetric optimization. Collectively, these advances have the potential to transform PBM from an empirical intervention into a reproducible, evidence-based therapeutic modality.

## 6 Functional quantification of laser stimulation through cellular response

The quantification of photobiomodulation (PBM) treatment requires intra- and intercellular biomarkers to assess both the immediate and long-term cellular responses in muscle tissue. Of these biomarkers, mitochondrial membrane potential, reactive oxygen species (ROS) synthesis, nitric oxide (NO) release, and calcium ion channel activity provide empirical metrics for assessing the effects of laser stimulation on skeletal muscle cells. This section examines the principal biomarkers and functional assays used to quantify PBM-induced cellular responses and to evaluate skeletal muscle regeneration across experimental models.

The process of skeletal muscle repair is defined by an initial phase of inflammatory response activation, followed by a phase of myocyte proliferation and differentiation (Chargé and Rudnicki, 2004).

This phase of proliferation and differentiation from immature myoblasts to mature myotubes is characterized by the expression of myoblast determination protein 1 (MyoD) and myosin heavy chain (MyHC) (Asakura and Kikyo, 2022). There is foundational evidence of the ability of PBM to upregulate transcription factors, including MyoD, MyHC, and Myogenin, for muscle cell differentiation. Parigi et al. (2025) observed a significant increase in the expression of MyoD in C2C12 myoblasts, following PBM treatment with an energy density of 4 J/cm^2^. MyoD and MyHC fluorescence assays may serve as a physiologically-relevant method for quantifying skeletal muscle proliferation and differentiation. A differentiation index (Asakura and Kikyo, 2022) can be defined as the percentage of nuclei existing in any MyHC positive cell and a fusion index can be defined as the percentage of nuclei existing in multinucleated MyHC positive cells.

Together, these indices provide complementary measures of myogenic progression and enable standardized comparisons of regenerative outcomes across experimental PBM studies. By combining molecular marker expression with quantitative cellular metrics, researchers can more objectively evaluate the effectiveness of different PBM protocols and facilitate reproducibility across preclinical investigations.

The regeneration and repair of skeletal muscle is also affected by mitochondrial metabolism. Silveira et al. (2009) demonstrated that PBM increased oxidative phosphorylation, specifically cytochrome c oxidase activity, within complexes I-IV of the electron transport chain (ETC), which correlated with increased mitochondrial energetics and accelerated muscle repair in mice with muscle injury. Furthermore, Ferraresi et al. (2016) established that PBM promotes mitochondrial bioenergetics through increased oxidative phosphorylation for adenosine triphosphate (ATP) synthesis. Laser stimulation increased the mitochondrial membrane potential—electron flux and proton pumping—within C2C12 myoblasts, resulting in increased ATP production, which was visualized with tetramethylrhodamine methyl ester (TMRM). TMRM and MitoTracker Red fluorescence assays may serve as a method for quantifying mitochondrial membrane potential for live-cell and fixated-cell dynamics, respectively.

The stimulation of mitochondrial metabolism also induces various cellular repair mechanisms, including cytokine release, fibroblast proliferation, macrophage activation, etc. Malavazzi et al. (2024) demonstrated that PBM significantly reduced gene expression of inflammatory cytokines, including interleukin 6 (IL-6) and tumor necrosis factor alpha (TNF-α), thereby promoting muscle repair. The associated increase in mitochondrial metabolism with PBM leads to the formation of ROS, which regulates pro-inflammatory (IL-1, IL-6, TNF-α) and anti-inflammatory (IL-10) cytokines as well as the synthesis of NO, which induces vasodilation via the soluble guanylyl cyclase receptor (sGC), thereby improving blood flow, leukocyte migration, and oxygen delivery (Hamblin et al., 2006). While the formation of ROS is required for the regulation of inflammation, a high concentration can have cytotoxic effects that impair cell viability. This biphasic relationship mirrors the dose-dependent responses discussed in Section 5, emphasizing that both laser parameters and downstream oxidative signaling must remain within an optimal therapeutic window to promote regeneration while minimizing oxidative damage. Consequently, the modulation of ROS is dependent on the concentration of antioxidant enzymes, including catalase (CAT), superoxide dismutase (SOD), and glutathione peroxidase (GPx). Ribeiro et al. (2016) demonstrated that PBM increased the activity of antioxidant enzymes, specifically CAT, SOD, and GPx. General Oxidative Stress Indicator (CM-H2DCFDA) and MitoSOX Green fluorescence assays may serve as a method for quantifying cellular and mitochondrial oxidative stress, respectively (Ribeiro et al., 2016). When interpreted alongside treatment parameters such as wavelength, irradiance, fluence, and exposure duration, these oxidative stress biomarkers provide an objective means of evaluating whether PBM protocols achieve the desired therapeutic window. Consequently, integrating dosimetric parameters with quantitative cellular biomarkers offers a more comprehensive framework for optimizing treatment protocols and improving reproducibility across experimental and clinical studies.

Beyond oxidative stress and mitochondrial bioenergetics, intracellular calcium functions as a critical secondary messenger that integrates PBM-induced photostimulation with downstream signaling pathways governing cell proliferation, differentiation, and tissue regeneration. Consequently, evaluating calcium dynamics provides an additional functional biomarker for assessing the biological effectiveness of PBM protocols. The regeneration and repair of skeletal muscle is also affected by intracellular calcium release. Tong et al. (2025) reported that PBM increased intracellular calcium levels in meniscus-derived stem cells, which resulted in the increase of ROS production, osteogenic cell differentiation, mitochondrial membrane potential, and cell viability. Additionally, an increase in intracellular calcium levels triggers calcium-sensitive and calmodulin-dependent enzymes, which amplify intracellular signaling cascades, allowing for increased regeneration and repair of skeletal muscle. A table consisting of fluorescence assays for quantification of laser stimulation of skeletal muscle is listed below (Table 1).

**TABLE 1:** Quantitative assays for assessing photobiomodulation (PBM) effects on skeletal muscle regeneration.

Assay (Antibodies, stains, etc.)	Manufacturer	Catalog number	Color	Target	Function
Myoblast Determination Protein 1 (MyoD) Antibody	Santa Cruz Biotechnology	sc-377186	Variable	MyoD Protein	Detects MyoD; distinguishes differentiating myotubes from immature myoblasts
Myosin Heavy Chain (MyHC) Antibody	Thermo Fisher Scientific Inc.	MA5-35613	Variable	Myosin Heavy Chain Protein	Detects MyHC; distinguishes differentiating myotubes from immature myoblasts
AlexaFluor 488 Donkey Anti-Mouse IgG Antibody	Thermo Fisher Scientific Inc.	A-21202	Green	Mouse IgG	Secondary antibody for visualization of primary antibodies bound to MyoD or MyHC
DAPI (4′,6-diamidino-2-phenylindole) Stain	Thermo Fisher Scientific Inc.	D1306	Blue	DNA	Binds DNA, allowing quantification of myonuclei
Tetramethylrhodamine Methyl Ester (TMRM) Stain	Thermo Fisher Scientific Inc.	T668	Red	Mitochondria (Potential-Dependent)	Assesses active mitochondrial membrane potential and activity
MitoTracker™ Green FM	Thermo Fisher Scientific Inc.	M7514	Green	Mitochondria (Potential-Independent)	Assesses mitochondrial content and mass
MitoTracker™ Red FM Stain	Thermo Fisher Scientific Inc.	M22425	Red	Mitochondria (Potential-Dependent)	Assesses mitochondrial membrane potential and activity
General Oxidative Stress Indicator (CM-H2DCFDA) Stain	Thermo Fisher Scientific Inc.	C6827	Green	General Reactive Oxygen Species (ROS)	Detects oxidative stress by measuring hydroxyl radicals, peroxyl radicals, and reactive oxygen species
MitoSOX™ Green Stain	Thermo Fisher Scientific Inc.	M36008	Green	Mitochondrial Superoxide	Detects mitochondrial oxidative stress by measuring superoxide production and reactive oxygen species
Fluo-8 AM Stain	Abcam Limited	ab142773	Green	Intracellular Calcium	Binds calcium, allowing measurement of muscle contraction

A comprehensive evaluation of the effects of PBM on skeletal muscle repair requires an integrated, multimodal approach that combines the use of these biomarkers with real-time analysis, which can be integrated with biomaterials. Beyond improving experimental reproducibility, standardized biomarker assessment has important implications for clinical translation. The integration of molecular biomarkers, fluorescence-based functional assays, and emerging computational approaches may enable patient-specific monitoring of treatment response and facilitate the development of precision PBM protocols. Such multimodal evaluation strategies will be essential for establishing objective therapeutic endpoints and accelerating the transition of PBM from experimental investigation to routine clinical practice.

## 7 Preclinical vs. clinical evidence

The translation of preclinical photobiomodulation (PBM) findings into reproducible clinical outcomes remains a persistent challenge. PBM has consistently demonstrated beneficial effects in animal and *in vitro* models, including enhanced muscle recovery, reduced oxidative stress, and increased mitochondrial activity (Assis et al., 2016). This disparity arises from both biological and methodological differences, including variations in tissue optical properties, irradiation geometry, metabolic environment, and outcome measurement techniques. A major source of this discrepancy lies in the scaling of optical properties between experimental models and human tissue. Yang et al. (2024) provided a comprehensive analysis of red and near-infrared light dosimetry, demonstrating that photon absorption and scattering are not linearly related to tissue composition and hydration. Animal tissues are generally thinner and less pigmented than human tissues, allowing deeper light penetration and higher effective irradiance at the target site, as demonstrated in rodent models (Sabino et al., 2016). Salehpour et al. (2019) extended these findings to cranial tissues, with implications for deeper tissues. Therefore, parameter values optimized in rodents may underdose human tissue at therapeutic depths unless compensated by increased power or exposure duration. This limitation illustrates that successful clinical translation depends not only on reproducing effective irradiation parameters but also on accounting for fundamental anatomical and physiological differences between experimental models and human tissues, as depicted in Figure 4.

**FIGURE 4:**
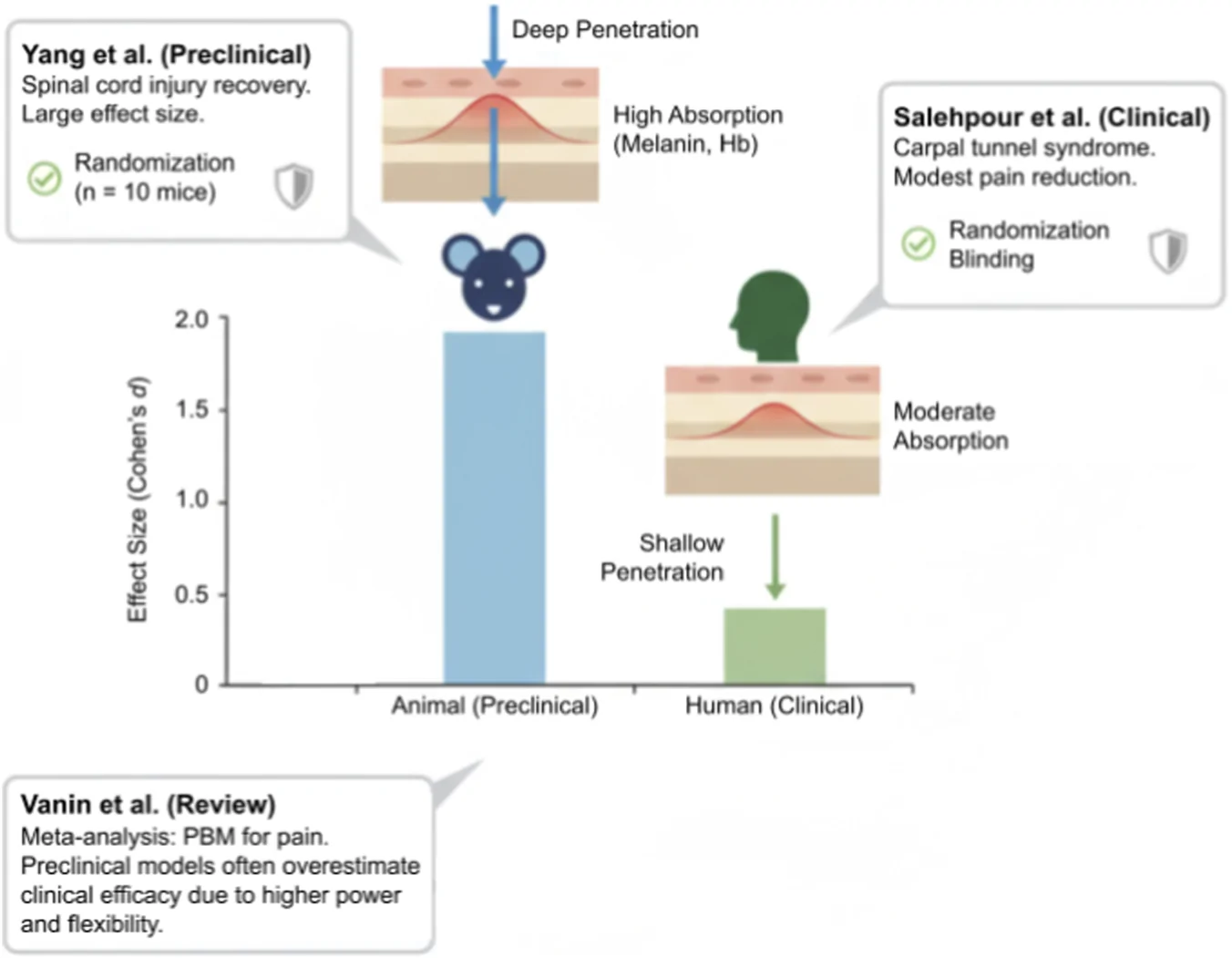
Comparison of Preclinical and Clinical Photobiomodulation Outcomes. Conceptual illustration of translational differences between preclinical and clinical photobiomodulation studies. The embedded labels are retained from the original conceptual graphic and are included for illustrative purposes only. They do not represent quantitative effect sizes, clinical outcomes, or direct findings from Yang et al. (2024) or Salehpour et al. (2019). This figure highlights the translational gap between controlled preclinical studies and heterogeneous clinical investigations, emphasizing the need for standardized dosimetry, rigorous reporting, and individualized treatment planning to improve the reproducibility and clinical translation of PBM.

Experimental rigor also varies significantly between model systems. Van der Worp and Macleod (2011) showed that low methodological quality, particularly inadequate randomization, lack of blinding, and small sample sizes, can inflate reported treatment effects. Similar biases are observed in PBM studies, where low statistical power and variable controls obscure true effect sizes. Consequently, discrepancies between preclinical and clinical findings may reflect differences in experimental design and reporting quality as much as true biological variation, reinforcing the importance of standardized methodological frameworks. Dyson and Singer (2009) noted that animal models, while mechanistically informative, often fail to capture the multifactorial nature of human pathophysiology, including systemic inflammation and comorbidities, thereby overestimating clinical benefits. Vanin et al. (2018) reported that PBM consistently improves fatigue resistance and muscle performance in both animal and human studies, although effect sizes are smaller in clinical settings. Razaghi et al. (2022) reported similar findings: while PBM improves implant stability and bone remodeling in animal models, human orthodontic trials show only modest or short-lived effects with the same dosimetry, reflecting species-specific healing differences. These findings highlight that photobiological parameters must be interpreted within their specific anatomical and metabolic contexts rather than being directly extrapolated across species. Lowenstein and Castro (2009) highlighted a broader translational crisis in biomedical science, where many phase III trials fail due to inadequately modeled human variability despite strong preclinical data. In addition, PBM outcomes are further influenced by interindividual variability in tissue optical properties and mitochondrial redox states. Sabino et al. (2016) modeled the optical properties of mouse skin and found that reflection and scattering coefficients differ significantly from those in humans, leading to inaccurate dose estimations. These optical, biological, and methodological differences explain why preclinical success does not translate linearly into clinical efficacy. Improving translational reliability will require the use of computational dosimetry and individualized treatment planning. This includes Monte Carlo–based light propagation modeling combined with patient-specific tissue properties to reduce the translational gap between animal and human tissues (Yang et al., 2024). Standardization of animal study reporting and prospective trial registration will further improve the predictive validity of preclinical PBM data. Only through standardized methodologies, physiologically realistic modeling, and patient-specific dosimetric optimization can PBM achieve consistent and reproducible clinical outcomes. Collectively, these advances will help bridge the gap between promising experimental findings and reliable evidence-based clinical implementation.

Table 2 highlights both the promise and the limitations of current clinical evidence. Across randomized controlled trials, PBM consistently improved muscle performance, reduced biochemical markers of muscle damage, and accelerated post-exercise recovery. However, considerable heterogeneity exists in irradiation parameters, including wavelength, delivered energy, device configuration, application timing, and outcome measures. Furthermore, most studies enrolled healthy athletes with relatively small sample sizes, limiting extrapolation to broader clinical populations. These methodological differences complicate direct comparisons between studies and emphasize the need for standardized dosimetric reporting and clinically validated treatment protocols.

**TABLE 2:** Summary of clinical studies evaluating photobiomodulation for skeletal muscle applications.

Author (Year)	Clinical condition	Sample size	Wavelength (nm)	Energy density (J/cm^2^)	Treatment protocol	Primary outcomes	Major limitations
Leal-Junior et al. (2009)	Exercise-induced skeletal muscle fatigue (volleyball athletes)	10	660/850 (LED cluster)	Device-specific total energy (41.7 J delivered)	PBMT immediately before repeated biceps contractions	Increased repetitions to fatigue and improved recovery biomarkers	Very small sample; elite athletes only; upper-limb model limits generalizability (Wiley Online Library)
Aver Vanin et al. (2016)	Post-exercise recovery in soccer players	28	810	10, 30, or 50 J dose (dose-response trial)	PBMT before eccentric quadriceps exercise	Improved MVC, reduced CK and IL-6, optimal response at 50 J; no significant effect on DOMS	Healthy athletes only; short-term follow-up; single-center study (Sage Journals)
de Oliveira et al. (2017)	Optimization of PBMT output power	28	810	10 J	PBMT before eccentric exercise using different power outputs	Improved muscle performance and reduced biochemical markers with 100–200 mW output	Focused on output power rather than broader clinical populations (Sage Journals)
Tomazoni et al. (2019)	Intense running performance in elite soccer players	22	810	Protocol-specific	PBMT before progressive running test	Improved exercise performance and reduced oxidative stress, inflammation, and muscle damage	Elite athletes only; crossover design may limit external generalizability (Wiley Online Library)
de Paiva et al. (2016)	Recovery after eccentric muscle damage	50	Multi-diode PBMT	Protocol-specific	PBMT, cryotherapy, combination therapy, or placebo	PBMT superior to cryotherapy for recovery of muscle function and biochemical markers	Healthy volunteers rather than injured patients; limited long-term outcomes (PubMed)

## 8 Practical guidelines for clinical application of photobiomodulation

Evidence from preclinical and clinical studies supports a key conclusion: photobiomodulation (PBM) should be individualized based on tissue depth, treatment objectives, and underlying pathology to achieve reliable outcomes. However, variability in reported parameters continues to obscure interpretation of findings, although existing reviews and clinical guidelines provide a foundation for standardization (Zecha et al., 2016; Zhang and Qu, 2023; Shivappa et al., 2025).

To facilitate clinical translation and improve consistency in parameter selection and reporting, Table 3 summarizes evidence-informed dosimetric considerations derived from current clinical guidelines, methodological recommendations, and recent multidisciplinary reviews of PBM (Shivappa et al., 2025). Because optimal PBM parameters remain dependent on tissue characteristics, treatment objectives, and individual patient factors, these values should be interpreted as reference ranges rather than universally standardized treatment prescriptions.

**TABLE 3:** Evidence-informed dosimetric considerations and reporting recommendations for clinical PBM.

Treatment consideration	Evidence-informed recommendation	Supporting evidence/Rationale
Target tissue depth	Select wavelength according to tissue depth	Tissue penetration increases with wavelength; deeper tissues require near-infrared irradiation
Superficial tissues	630–660 nm, approximately 2–4 J/cm^2^	Effective for superficial tissues such as skin and oral mucosa (Zhang and Qu, 2023)
Deep tissues (skeletal muscle)	810–980 nm, approximately 4–8 J/cm^2^	Greater penetration allows therapeutic energy delivery to skeletal muscle and deeper musculoskeletal tissues (Zhang and Qu, 2023)
Irradiation mode	Continuous-wave irradiation is commonly used; pulsed delivery (10–100 Hz) may be considered for selected regenerative applications	Evidence for pulsed delivery remains promising but has not been fully standardized
Essential dosimetric reporting	Report wavelength, output power, beam area, irradiation time, energy, fluence (energy density), pulse characteristics (if applicable), treatment location, number of treatments, and treatment interval	Recommended by Jenkins and Carroll (Jenkins and Carroll, 2011) and reinforced by Hadis et al. (2016) to improve reproducibility and comparison among studies
Device quality assurance	Perform radiometric calibration and verify actual optical output rather than relying solely on manufacturer specifications	Zecha et al. (2016) and Hadis et al. (2016) emphasize calibration and standardized dosimetry as prerequisites for reproducible clinical implementation
Clinical implementation	PBM should be integrated with evidence-based rehabilitation and individualized according to tissue characteristics, treatment goals, and patient-specific factors	Current evidence supports individualized treatment planning rather than universal protocols

Zhang and Qu (2023) emphasize that optimal PBM parameters depend on tissue optical penetration depth. Superficial tissues, such as skin, oral mucosa, and joints, typically respond to red light (630–660 nm) at commonly reported fluences of approximately 2–4 J/cm^2^ which effectively modulate mitochondrial activity and inflammation. Deeper tissues, including skeletal muscle and joint capsules, generally require near-infrared wavelengths (810–980 nm) and fluences of approximately 4–8 J/cm^2^ to achieve therapeutic effects. Continuous-wave irradiation is typically used for anti-inflammatory and analgesic effects. In contrast, in some studies, pulsed light (10–100 Hz) has been proposed to enhance oxygen availability and promote regenerative signaling through intermittent mitochondrial reoxygenation.

Clinical translation of PBM has been most successful in oncology and anti-inflammatory applications, where precise parameter control has enabled reproducible outcomes. Zecha et al. (2016) established standardized PBM protocols for preventing oral mucositis in patients undergoing chemoradiation, demonstrating that controlled dosimetry and accurate energy delivery can produce reproducible clinical benefits. Their work highlights three key principles applicable to musculoskeletal rehabilitation: (i) accurate dosing per unit area, (ii) appropriately spaced treatment intervals to prevent biological adaptation, and (iii) device calibration using radiometric validation. These principles demonstrate how standardized protocols can transform PBM from an empirical intervention into a predictable therapeutic modality. Importantly, these principles are equally applicable to skeletal muscle PBM, where consistent dosimetric reporting and protocol standardization remain essential for improving reproducibility, facilitating cross-study comparisons, and supporting broader clinical adoption.

Clinical efficacy is further enhanced when PBM is used adjunctively with conventional rehabilitation strategies. Rather than functioning as a replacement for established rehabilitation approaches, PBM should be considered an adjunctive therapy that enhances physiological recovery when integrated within evidence-based clinical management. When combined with exercise or physiotherapy, PBM enhances physiological responses (particularly in skeletal muscle) by supporting mitochondrial biogenesis, reducing oxidative stress, and accelerating tissue repair (Vanin et al., 2018; Zhang and Qu, 2023). This synergistic approach, integrating PBM with mechanical loading and conventional rehabilitation, provides a reproducible framework for post-surgical recovery, sports medicine, and musculoskeletal rehabilitation. For clinical implementation, all PBM studies must clearly report core dosimetric parameters, including wavelength, beam area, power output, energy density, pulse frequency, treatment duration, and timing relative to injury or inflammation. Zecha et al. (2016) demonstrated that adherence to standardized reporting improves consistency and safety in clinical applications. These principles are directly translatable to musculoskeletal PBM, where addressing inconsistencies in reporting would improve reproducibility, clinical applicability, and regulatory acceptance. Collectively, these practical recommendations highlight that successful clinical implementation of PBM depends not only on selecting appropriate irradiation parameters but also on standardized treatment protocols, rigorous dosimetric reporting, and integration with established rehabilitation strategies. Continued refinement of these evidence-based guidelines will be essential for translating promising experimental findings into consistent, safe, and effective patient care.

## 9 Limitations in current photobiomodulation research

Despite strong mechanistic evidence and promising outcome data, photobiomodulation (PBM) research suffers from reproducibility issues that hinder translation into standardized therapeutic practice. The most common limitation across the literature is the incomplete or inconsistent reporting of key dosimetric parameters such as wavelength, energy density, beam profile, and exposure time. Draeger et al. (2020) supplied a meta-analysis of two decades of radiobiology studies, revealing that fewer than one-third of studies provided sufficient reporting of physical parameters to enable replication. This poor reporting has contributed to a “reproducibility gap,” in which similar protocols yield different outcomes due to unreported or inconsistently described optical delivery conditions, tissue geometries, or irradiance calibration methods. Importantly, these limitations are not independent challenges but interconnected factors that collectively reduce reproducibility and complicate comparisons across studies. Incomplete dosimetric reporting, inconsistent device calibration, and methodological heterogeneity each contribute to uncertainty in treatment efficacy, underscoring the need for standardized experimental and clinical reporting frameworks. Accurate radiometric quantification of the delivery of light remains a technical bottleneck. Hadis et al. (2016) have suggested that radiometry in low-level light therapy remains poorly standardized, noting that commercial power meters and LED array readouts are often neither calibrated nor wavelength-compensated, and are unable to accurately measure beam divergence. Calibration errors and detector misalignment can lead to fluence measurement inaccuracies exceeding 30%, introducing systematic error into reported dose–response relationships. These observations further emphasize that accurate radiometric calibration is fundamental to reproducible PBM research, as even minor measurement errors may substantially alter the delivered biological dose and confound interpretation of therapeutic outcomes. These measurement uncertainties propagate into clinical variability, complicating the establishment of reliable dose–response relationships and therapeutic dosing windows. Another error of variable reporting follows on account of the diversity of devices and optical geometries employed in the literature. Fernandes et al. (2024) have conducted a systematic review of PBM devices designed for brain photobiomodulation with results that show considerable variations in energy distribution, optical coupling, and thermal dissipation. Many commercial devices are not reported to be calibrated “conventionally,” leading to significant inter-device and inter-study variability. Parker et al. (2019) found that many dental PBM studies failed to report spot size, irradiance uniformity, or treatment distance, parameters critical to energy distribution and biological response. Equally problematic is the absence of universal reporting conventions in respect of PBM studies in the experimental and clinical settings. Jenkins and Carroll (2011) have suggested that some form of structured dose-reporting process would be useful, stressing the importance of reporting all optical and geometrical parameters along with biological outcomes. Standardized reporting guidelines would also facilitate higher-quality systematic reviews and meta-analyses by enabling meaningful comparisons across studies employing different devices, treatment protocols, and biological models. However, adoption of such guidelines remains inconsistent across journals and clinical trials. Without standardized reporting templates, meta-analyses and systematic reviews cannot reliably determine dose–response relationships or adequately account for device variability. Last, the absence of a cross-validation between laboratories has given rise to a dearth of consensus in respect of optimal dosage windows or therapeutic indices employed in the literature. Few studies have included inter-laboratory calibration or multi-centre designs using standardized PBM hardware, which would substantially improve reproducibility and clinical confidence. Correcting these methodological errors will require cooperative thinking with the development of uniform radiometry and reporting protocols, as well as the establishment of common databases of valid dosage-response tables. Until these methodological limitations are systematically addressed, PBM will continue to demonstrate considerable biological promise while facing challenges in achieving consistent clinical translation. Coordinated efforts toward standardized dosimetry, rigorous radiometric validation, multicenter collaboration, and transparent reporting will be essential for establishing PBM as a reproducible and evidence-based therapeutic modality.

## 10 Future directions in personalized and computational photobiomodulation

The future of photobiomodulation (PBM) research will likely involve integrating computational modeling, accurate dosimetry, and personalized therapy to overcome longstanding limitations in reproducibility and clinical translation. Current experimental and clinical approaches largely rely on empirical parameter selection, resulting in variability between studies and devices. The next-generation of PBM research is expected to incorporate physics-based and data-driven methodologies, enabling dose prediction and patient-specific optimization based on anatomy and tissue optical properties (Yaroslavsky et al., 2021).

### 10.1 Comparison of Monte Carlo modeling with alternative light transport models

*How do different light transport models compare?* Several computational approaches have been developed to model light propagation in biological tissues, each offering distinct advantages and limitations. Among the simplest is the Beer–Lambert law, which estimates light attenuation as an exponential function of tissue thickness and absorption. Although computationally efficient, this approach neglects photon scattering and assumes a homogeneous optical medium, making it unsuitable for accurately predicting light transport in skeletal muscle, where multiple tissue layers and heterogeneous optical properties substantially influence photon distribution.

The diffusion approximation provides a more sophisticated description of photon transport by incorporating scattering effects while remaining computationally efficient. It performs well in highly scattering tissues and at locations distant from the light source but loses accuracy near tissue boundaries, superficial irradiation sites, and regions with low scattering or strong absorption. Consequently, its applicability for modeling localized photobiomodulation treatments is limited.

The radiative transfer equation (RTE) represents the most complete mathematical description of photon propagation by explicitly accounting for both absorption and scattering. However, solving the RTE directly for anatomically realistic biological tissues is computationally demanding and often impractical for routine clinical treatment planning. Numerical implementations based on finite element or finite difference methods partially address this challenge by discretizing tissue geometries and boundary conditions. While these approaches improve computational efficiency, they generally rely on simplifying assumptions regarding tissue optical properties and may lose accuracy when applied to highly heterogeneous biological structures.

Monte Carlo (MC) simulation remains the reference standard for modeling light transport in biological tissues because it statistically simulates the trajectories of individual photons while accounting for multiple scattering, absorption, reflection, refraction, and complex tissue geometries. Unlike analytical or diffusion-based approaches, MC methods readily incorporate multilayer anatomical models and patient-specific optical properties (Yaroslavsky et al., 2021), providing highly accurate estimates of internal light distribution. Although computationally intensive, recent advances in graphical processing unit (GPU) acceleration have substantially reduced simulation times, making anatomically realistic MC modeling increasingly feasible (Yaroslavsky et al., 2021) for both research and translational applications.

For skeletal muscle photobiomodulation, where tissue heterogeneity, anisotropic fiber architecture, vascularization, and patient-specific optical properties strongly influence photon propagation, MC simulation currently provides the most physiologically realistic framework for dosimetric optimization. Consequently, it serves as the benchmark against which alternative light transport models are evaluated and represents the most suitable computational foundation for future precision photobiomodulation strategies (Yaroslavsky et al., 2021).

### 10.2 Patient-specific computational modeling

*How can we personalize Monte Carlo modeling?* Building upon these foundational developments, Monte Carlo modeling has evolved from simplified multilayer tissue representations toward anatomically realistic, patient-specific simulations. Advances in graphical processing unit (GPU) computing have substantially improved computational efficiency, enabling high-resolution models that better capture the optical heterogeneity of skeletal muscle and surrounding tissues. These developments have significantly enhanced the feasibility of Monte Carlo–based dosimetric optimization for both preclinical research and future clinical applications.

Recent studies have demonstrated the feasibility of applying anatomically realistic Monte Carlo models to photobiomodulation dosimetry. Yaroslavsky et al. (2021) validated a Monte Carlo–based dosimetry framework for extraoral PBM by calibrating device output against theoretically derived energy distributions using magnetic resonance imaging (MRI)-based anatomical models. This work demonstrated the potential of integrating patient-specific anatomical information into treatment planning, representing an important step toward personalized PBM dosimetry. Similarly, Chen Z. et al. (2025) developed a high-resolution Monte Carlo simulation framework using a detailed rat atlas to investigate light propagation in a spinal cord injury model. Although focused on central nervous system applications rather than skeletal muscle, the study demonstrated the value of anatomically realistic tissue modeling and reported that 1,064 nm achieved the greatest penetration depth and energy delivery to target tissues compared with 660, 808, and 980 nm. Collectively, these studies illustrate the ongoing evolution of Monte Carlo modeling from simplified optical simulations toward clinically relevant, anatomically informed computational platforms capable of supporting precision photobiomodulation.

Patient-specific biophysical modeling represents another promising direction for advancing personalized photobiomodulation. Le et al. (2017) pioneered computational modeling frameworks for tumor growth and radiotherapy treatment planning that integrate imaging data and predictive algorithms for therapy personalization. Although originally developed for oncology applications, this methodology could be adapted for PBM to generate individualized fluence profiles based on tissue thickness, melanin content, and vascular characteristics Dong et al. (2025) demonstrated this concept through patient-specific modeling using optical diffusivity to simulate near-infrared light therapy for Alzheimer’s disease. Their findings highlight how inter-patient variability in skull thickness and tissue optical properties significantly affects photon distribution. More recent work by Yaroslavsky et al. (2023) aimed to optimize extraoral PBM parameters for mucosal healing (but not muscle). Importantly, they took a personalized approach and coupled archival MRI imaging with patient-specific MC modeling. Collectively, these studies highlight the potential of patient-specific computational modeling to support individualized PBM treatment planning for musculoskeletal applications.

### 10.3 Artificial intelligence–assisted PBM optimization

*How can AI enhance computational modeling?* Despite its high accuracy, Monte Carlo simulation remains computationally demanding and is not well suited for real-time implementation or inverse problem solving because of its computational cost and stochastic convergence characteristics. This is a challenge for using MC in PBM for determining tissue properties (inverse problem) and when tracking the muscle regeneration and optimizing parameters through therapy. Furthermore, modeling specific cases such as muscle regeneration requires higher resolution modeling, which is difficult as it is computationally intensive. Artificial intelligence in the form of machine/deep learning has recently become a viable option to address these issues by augmenting or partially replacing MC. Hokr and Bixler trained a 5-layer fully connected neural network for inverting a MC simulation to extract tissue properties (Hokr and Bixler, 2021). Furthermore, they also proposed an experimental setup *in vivo* and in real time to further improve the accuracy of tissue optical property measurements. Ardakani et al. used a Convolutional Neural Network (CNN) to denoise low-photon MC simulations (Raayai Ardakani et al., 2022). The noise reduction provided simulation results in par with high-photon count MC simulations, significantly speeding up the MC simulation times. Similarly, Fu et al. used a Fourier Neural Network (FNN) for low-photon MC simulations, and reduced the required photon count by 3 orders of magnitude (Fu et al., 2025). Artificial intelligence has the potential to substantially enhance the efficiency and clinical applicability of Monte Carlo–based dosimetry by accelerating simulations and improving tissue property estimation. However, robustness of these systems requires further research by enhancing the datasets with real tissue data, and by addressing the broader issues in AI such as interpretability and generalizability. Importantly, artificial intelligence should be viewed as a complementary tool that enhances computational efficiency and predictive capability rather than replacing mechanistic understanding or experimental validation. Continued integration of AI with optical physics, biological experimentation, and clinical evidence will be essential for developing reliable precision PBM platforms.

### 10.4 Adaptive precision photobiomodulation

*What could future clinical PBM systems look like?* The convergence of advanced computational modeling and artificial intelligence naturally supports the development of adaptive treatment systems capable of responding to patient-specific physiological feedback in real time. Computational tools such as Monte Carlo simulation, combined with real-time physiological feedback systems and artificial intelligence, could enable the next-generation of adaptive PBM dosing. By integrating wearable sensors and imaging modalities such as EMG and fNIRS, fluence or pulse frequency could be dynamically adjusted based on physiological responses. Such closed-loop systems have the potential to transform PBM from a static light-delivery modality into an adaptive precision therapy that continuously adjusts treatment parameters according to patient-specific physiological responses. Ultimately, the integration of Monte Carlo modeling, patient-specific biophysical modeling, wearable physiological monitoring, and artificial intelligence–driven optimization has the potential to transform PBM from an empirically guided intervention into a precision-based therapeutic platform. Achieving this vision will require continued interdisciplinary collaboration among engineers, physicists, biologists, clinicians, and regulatory bodies to ensure that technological innovation translates into safe, reproducible, and clinically meaningful patient outcomes.

## 11 Conclusion

Photobiomodulation (PBM) has emerged as a promising non-invasive therapeutic modality for enhancing skeletal muscle performance, accelerating tissue repair, and supporting functional recovery through mechanisms involving mitochondrial bioenergetics, modulation of oxidative stress, regulation of inflammation, and activation of regenerative signaling pathways. Collectively, the preclinical and clinical evidence reviewed in this manuscript indicates that PBM has considerable therapeutic potential across a broad range of musculoskeletal applications.

Despite these encouraging findings, widespread clinical implementation remains constrained by substantial heterogeneity in treatment protocols, inconsistent reporting of dosimetric parameters, and limited standardization of irradiation methods. Variations in wavelength selection, irradiance, fluence, treatment timing, device characteristics, and outcome measures continue to complicate comparisons among studies and hinder the establishment of evidence-based clinical guidelines. Future investigations should therefore prioritize rigorous dosimetric reporting, radiometric calibration, standardized treatment protocols, and well-designed multicenter clinical trials to improve reproducibility and facilitate clinical translation.

Advances in computational modeling, particularly Monte Carlo–based light transport simulations, together with patient-specific anatomical modeling and emerging artificial intelligence–assisted optimization strategies, provide new opportunities to individualize PBM treatment planning and improve dosimetric precision. These computational approaches have the potential to bridge the gap between experimental photobiology and personalized clinical therapy by enabling more accurate prediction of photon distribution within heterogeneous musculoskeletal tissues.

Overall, PBM demonstrates considerable promise for improving skeletal muscle performance, tissue repair, and functional recovery. However, several important barriers continue to limit its widespread clinical implementation, including the lack of consensus dosimetry reporting standards, inconsistent radiometric calibration of commercial devices, limited inter-laboratory validation, the absence of clearly defined regulatory pathways, and the challenges of translating optical dosimetry from animal models to human tissues. Addressing these limitations through standardized reporting, rigorous device calibration, multicenter validation studies, and clinically relevant computational modeling will be essential before PBM can achieve consistent, reproducible, and evidence-based implementation in musculoskeletal medicine.
